# Comparison of Retinal Microvascular Changes in Axial Spondyloarthritis Using Optical Coherence Tomography Angiography: Anti-TNF vs. NSAID Therapy

**DOI:** 10.3390/diagnostics15050597

**Published:** 2025-03-01

**Authors:** Özlem Karataş, Çisil Erkan Pota, Bülent Akyüz, Yusuf Samet Atlıhan, Kaan Pota, Aslı Çetinkaya Yaprak, Merve Sarı, Serpil Tuna

**Affiliations:** 1 Department of Physical Medicine and Rehabilitation, Akdeniz University, Antalya 07058, Turkey; drbulentakyuz@outlook.com (B.A.); serpiltuna@akdeniz.edu.tr (S.T.); 2 Department of Ophthalmology, Faculty of Medicine, Akdeniz University, Antalya 07058, Turkey; cisilerkann@gmail.com (Ç.E.P.); ysf20smt@gmail.com (Y.S.A.); aslickaya@yahoo.com (A.Ç.Y.); 3 Department of Orthopaedics and Traumatology, Akdeniz University, Antalya 07058, Turkey; kaanpota@hotmail.com; 4 Department of Internal Medicine, Antalya Training and Research Hospital, Antalya 07058, Turkey; mervee-sari@hotmail.com

**Keywords:** axial spondyloarthritis, anti-TNF therapy, NSAIDs, swept-source optical coherence tomography angiography, retinal microvascular changes, choroidal thickness, vascular density

## Abstract

**Objectives**: The aim of this study was to investigate retinal and choroidal microvascular changes in patients with axial spondyloarthritis (axSpA) treated with long-term anti-TNF therapy and NSAIDs and in healthy control subjects using optical coherence tomography angiography (SS-OCT-A). **Methods**: A total of 162 eyes from 81 participants were included: 52 eyes from 26 axSpA patients treated with anti-TNF therapy (≥5 years), 44 eyes from 22 axSpA patients treated with NSAIDs, and 66 eyes from 33 healthy control subjects. SS-OCT-A imaging was used to assess retinal thickness, ganglion cell layer thickness, retinal nerve fiber layer thickness, and the vessel densities of the superficial capillary plexus (SCP), deep capillary plexus (DCP), and choriocapillaris (CC). Disease activity was assessed with ASDAS-CRP. **Results**: Both axSpA subgroups showed a significant expansion of the foveal avascular zone and reduced SCP and DCP densities compared to the controls. The CC vessel density was higher in axSpA patients than in healthy subjects. The anti-TNF group had a lower CC vascular density than the NSAIDs group. The disease duration correlated with a decreased central DCP density and increased paracentral SCP and CC densities. **Conclusions**: SS-OCT-A revealed subclinical retinal and choroidal changes in axSpA patients, highlighting the impact of chronic inflammation on the retinal vasculature. While anti-TNF therapy effectively controls systemic inflammation, it cannot completely prevent microvascular changes. Further studies are needed to assess the clinical relevance of these results.

## 1. Introduction

Axial spondyloarthritis (axSpA) is a chronic inflammatory disease that primarily affects the spine and sacroiliac joints [1]. Due to the presence of systemic inflammation, a significant proportion of patients have extra-articular manifestations such as ocular, cardiovascular, and gastrointestinal involvement [2]. Although anterior uveitis is the most commonly reported ocular involvement, the potential impact of chronic inflammation on retinal microvascular structures in axSpA is not yet fully understood. The chronic course of inflammation, which may be associated with endothelial dysfunction and the development of subclinical vasculitis, suggests that the identification of alterations in the retinal microcirculation may be an important step towards a deeper understanding of the disease [3].

Optical coherence tomography angiography (OCT-A) is a non-invasive method that can be used to visualize retinal microvascular structures with a high resolution. In recent years, it has gained popularity, particularly because of its potential to detect subclinical vascular changes in rheumatologic and autoimmune diseases [4]. In the literature, a limited number of studies using OCT-A in patients with axSpA have focused on comparing changes in retinal vessel density and perfusion parameters with healthy controls [5,6]. Therefore, there are still no consistent conclusions on the effects of different treatment modalities, especially anti-TNF agents and NSAIDs, on retinal microvascular structures.

Anti-TNF agents, which have been shown to be effective in the treatment of axSpA, are thought to affect vascular structures by suppressing systemic inflammation. However, there are insufficient data on the extent to which this improvement translates into measurable microvascular changes at the retinal level [7]. While NSAIDs are known to have limited effects on controlling symptoms and reducing disease activity, their long-term impact on the degree of axial inflammation and consequently on the retinal microcirculation is still unclear [8].

With this in mind, the primary aim of this study was to investigate and compare retinal microvascular structures in axSpA patients receiving anti-TNF therapy, axSpA patients treated with NSAIDs alone, and a healthy control group using OCT-A. In particular, this study was designed to determine whether treatment-related microvascular changes occur and, if present, to investigate their association with subclinical vascular changes. The knowledge acquired can make an important contribution to the early detection of microvascular changes in axSpA patients and to the development of follow-up protocols.

## 2. Material and Methods

This interdisciplinary, single-center study was conducted with the approval of the local ethics committee of Akdeniz University (approval number: 19 July 2023/KAEK 552). All procedures were in accordance with the ethical guidelines specified in the Declaration of Helsinki. Prior to participation in the study, written informed consent was obtained from all individuals participating in the study. The patient group was selected from volunteers diagnosed with axSpA according to the classification criteria of the Assessment of Spondylo Arthritis International Society (ASAS) and followed up at the Rheumatology Clinic [9]. The control group comprised patients without additional diseases who presented to the eye clinic for a routine eye examination and agreed to participate in the study.

The exclusion criteria were applied uniformly to all groups. Subjects with a history of systemic diseases such as diabetes mellitus or hypertension, previous ocular surgeries, or diseases such as uveitis, glaucoma, cataracts, or retinal diseases that could affect visual acuity were excluded. In addition, participants who had taken ophthalmic medication in the past, smoked, or had a refractive error of more than 3 diopters were not included in the study. Initially, 61 patients with axSpA were screened for eligibility. Of these, 13 patients (21.3%) were excluded due to a history of uveitis according to our exclusion criteria. To exclude the possible influence of vascular parameters influenced by the degree of ocular involvement and to rule out deterioration of image quality in uveitic patients (e.g., distortion of imaging caused by posterior synechiae or cataracts caused by topical steroids in uveitis), patients with other systemic diseases, previous ocular surgery, or retinal diseases were also excluded.

After selecting the patients, we started taking measurements. Ten eyes were excluded because they had a spherical refractive error of more than four diopters, cataracts, or amblyopia. In addition, 4 eyes were excluded due to insufficient image quality. Thus, the study included a total of 162 eyes: 52 eyes of 26 axSpA patients in the an-ti-TNF group, 44 eyes of 22 axSpA patients in the NSAIDs group, and 66 eyes of 33 healthy individuals in the control group.

The patient group was divided into two subgroups: the anti-TNF group and the NSAIDs group. The anti-TNF group consisted of patients who had received anti-TNF therapy for at least 5 years and had no systemic diseases other than axSpA. The anti-TNF agents used in this study included infliximab, adalimumab, etanercept, certolizumab, and golimumab. The NSAID group included people who were treated exclusively with non-steroidal anti-inflammatory drugs and who also had no systemic diseases other than axSpA.

Each participant underwent a comprehensive ophthalmologic examination, which included measurement of refractive error with an autorefractor (Nidek ARK-700A, Gamagori, Japan), determination of intraocular pressure (IOP) with a non-contact tonometer (NIDEK NT-2000, Gamagori, Japan), and assessment of best corrected visual acuity (BCVA). Both anterior and posterior segment examinations were performed using a slit-lamp biomicroscope. Imaging of the retina was performed using optical coherence tomography (SS-OCT) and optical coherence tomography angiography (SS-OCT-A) with the DRI OCT Triton system (Topcon, Tokyo, Japan). In addition, axial length (AL) measurements were performed with an optical biometer (IOLMASTER 500, Carl Zeiss, Jena, Germany).

### 2.1. Diagnosis and Follow-Up of AxSpA

The patients included in the study were diagnosed with axSpA according to the criteria of the Assessment of Spondylo Arthritis International Society (ASAS) [9]. HLA-B27 status, radiographic involvement, and disease duration were also assessed, and their relationships to retinal microvascular changes were analyzed.

### 2.2. Assessment of Disease Activity: ASDAS-CRP

Disease activity was objectively measured using the Ankylosing Spondylitis Disease Activity Score—C-Reactive Protein (ASDAS-CRP) [10]. ASDAS-CRP is a comprehensive scoring system used specifically to assess disease activity in axSpA patients. This score evaluates the patient’s inflammatory status and symptom severity by taking into account clinical manifestations and CRP levels. It is calculated based on symptoms such as fatigue, spinal pain, joint pain/swelling, local tenderness, and morning stiffness, as well as laboratory data. High ASDAS-CRP values indicate increased inflammation and active disease, while low values indicate well-controlled disease activity.

In this study, disease activity was graded using ASDAS-CRP scores and the correlations between these scores and changes in retinal microvascular structure were investigated. Furthermore, correlations between HLA-B27 positivity, disease duration, radiologic involvement, and retinal microvascular parameters were investigated.

### 2.3. Optical Coherence Tomography Angiography

OCT-A enables the acquisition of 3 × 3 en face images of the retinal vessels and facilitates the assessment of the superficial capillary plexus (SCP), the deep capillary plexus (DCP), and the choricapillaris (CC) vascular density (VD). The device enables precise measurement of the foveal avascular zone (FAZ), retinal and choroidal thickness (CT), and vascular density (VD) using SS-OCT (DRI OCT Triton; Topcon, Tokyo, Japan). It operates with a digital resolution of 2.6 µm, an axial resolution of 8 µm, a wavelength of 1050 nm, and a scanning speed of 100,000 scans per second. The analysis was performed with the IMAGEnet 6 software (version 1.24.1.15742) and the 3 × 3 mm SS-OCT angiogram tool.

**Figure 1:**
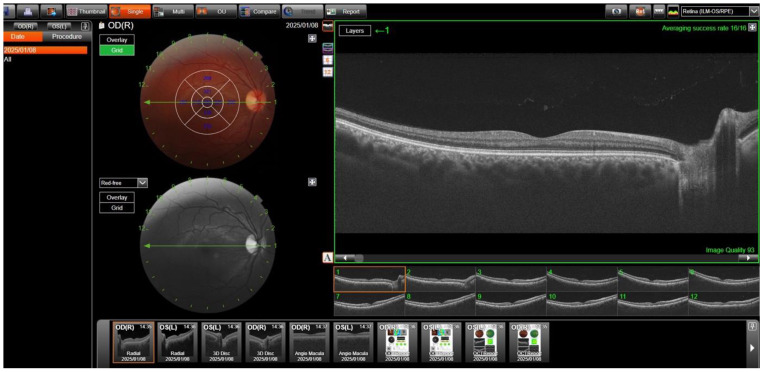
A representative illustration of OCT measurements: retinal, retinal nerve fiber layer, ganglion cell layer, and choroidal thickness.

**Figure 2:**
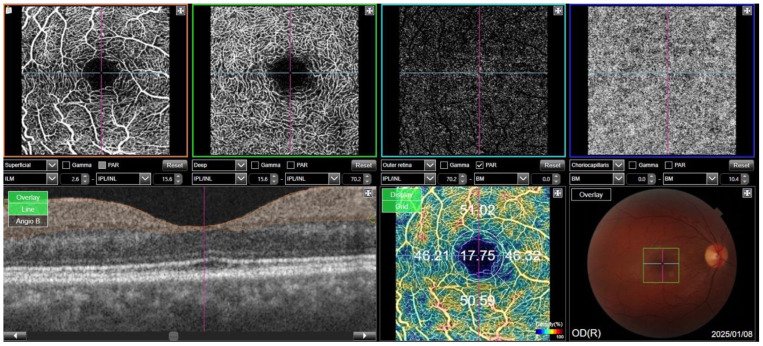
A representative illustration of OCTA measurements. The internal and external boundaries entered were used to determine the superficial, deep capillary plexus, and choriocapillaris vascular density values.

For the measurements, the central region was defined as the fovea-centered ring with a diameter of 1 mm, with the ring with a diameter of 3 mm around it representing the parafoveal region. This parafoveal region was divided into four quadrants: inferior, superior, temporal, and nasal. The software automatically measured choroidal thickness, central macular thickness, ganglion cell layer (GCL) thickness, and retinal nerve fiber layer (RNFL) thickness (Figure 1). All vascular density values for SCP, DCP, and choroidal blood flow (CC VD) were also measured automatically (Figure 2). However, the Topcon device (DRI OCT Triton) does not provide automatic calculation of the FAZ area. Instead, the FAZ area was measured manually by selecting the “Area” option, marking the boundary of the avascular zone and allowing the device to calculate the area of the marked zone.

### 2.4. Statistical Analysis

The data obtained within the scope of the research were analyzed using SPSS 26.0 (Statistical Package for the Social Sciences 26.0) and transferred to electronic media. The Kolmogorov–Smirnov test was used to analyze the normality of sample distribution. To define the sample, normally distributed values are presented as means ± standard deviation, and non-normally distributed values are presented as median (minimum–maximum). When comparing group means of variables with three or more categories, the ANOVA test was used in parametric conditions and the Kruskal–Wallis test was used in non-parametric conditions. Chi-square test was used to compare categorical data. Mann–Whitney U test was applied for subgroup analysis. Bonferroni correction and Tukey’s HSD were applied as posthoc tests. For the analysis of correlation, the Spearman correlation coefficient was used. A level of *p* < 0.05 was accepted as statistically significant.

## 3. Results

Patient demographics, IOP measurements, SE measurements, axial length measurements, ASDAS-CRP score, HLA B27 positivity, radiography positivity, and the duration of medication use are shown in Table 1. No significant difference was found between the groups with regard to the mean age of the patients. There were 24 (92.3%) male and 2 (7.7%) female individuals in the anti-TNF group, 7 (31.8%) male and 15 (68.2%) female individuals in the NSAID group, and 15 (45.45%) male and 18 (54.55%) female individuals in the control group. The number of male patients was statistically significantly higher in the anti-TNF group (*p* < 0.01). The presence of HLA B27 positivity and radiologic involvement was statistically significantly higher in the anti-TNF group. The ASDAS-Crp values were one in sixteen patients, two in five patients, three in three patients, and four in two patients in the anti-TNF group. These values were one in thirteen patients, two in seven patients, and three in two patients in the NSAID group. The OCT data are summarized in Table 2 and Table 3. The central retinal thickness and paracentral temporal retinal thickness were statistically significantly higher in the anti-TNF group than in the other groups (*p* = 0.044). The mean, nasal, and inferior RNFL measurements were significantly higher in the NSAIDs group (*p* = 0.024). The OCT-A results are summarized in Table 4. There was a statistically significant increase in FAZ area in the anti-TNF and NSAIDs group compared to the control group. No significant difference was found when the anti-TNF and NSAID groups were compared with each other (*p* = 0.542). There was a statistically significant decrease in SCP central VD values in the anti-TNF group compared to the other groups (*p* = 0.033). There was a statistically significant increase in SCP paracentral superior, inferior, and mean VD values in the anti-TNF and NSAID groups compared to the control group (*p* < 0.001). There was a decrease in the central and temporal VD values of the DCP in the anti-TNF and NSAID groups compared to the control group; there was a statistically significant increase in the superior and inferior VD values (*p* < 0.001). Choriocapillaris in all central and paracentral quadrants was statistically significantly increased in the anti-TNF and NSAID groups compared to the control group.

In the subgroup analysis, the VD values of SCP and DCP were lower in the anti-TNF group compared to the NSAID group, but the difference was not statistically significant (*p* > 0.05). The mean VD value of CC was statistically significantly lower in the anti-TNF group than in the NSAIDs group.

When we compared the patients by grouping all axSpA patients according to HLA B27 positivity, we found no significant difference in retina, choroidal thickness, or OCT-A values.

With increasing disease duration, we observed a decrease in the central VD of the DCP (*p* = 0.001, r = −0.468) and an increase in vessel density in the DCP paracentral, SCP paracentral, mean VD, and CC in all quadrants (*p* < 0.001, r = 0.451). We found no effect of the duration of drug use on the vascular parameters.

When we compared the ASDAS-CRP score with the OCT-A findings, we found no significant correlation between the scores and the vascular parameters.

## 4. Discussion

In this study, the retinal microvascular structures of axSpA patients receiving anti-TNF therapy, those taking NSAIDs, and those of the healthy control group were compared, and significant differences between the groups were found. We observed an increase in the FAZ area and a decrease in the central vessel densities of SCP and DCP in both groups compared to the healthy control group, regardless of the medication used in patients with axSpA. The CC VD was higher in patients with axSpA than in the control group. We observed a decrease in the central vascular density of DCP and an increase in other vascular parameters with increasing disease duration. The disease severity (ASDAS-CRP) and duration of medication had no effect on vascular parameters. The findings suggest that chronic inflammation in axSpA may have subclinical effects on retinal microvascular structures.

These findings support the negative effects of chronic inflammation on the retinal microcirculation. To date, few studies in the literature have examined microvascular changes in axSpA, and our results suggest that retinal microvascular changes may reflect the chronic inflammation in axSpA [3,11,12,13].

AxSpA is a multisystemic disease that primarily affects the axial skeletal system, but inflammatory processes can also have a significant impact on the systemic circulation and vascular network [14]. In AxSpA, the spread of inflammation to the vessel wall triggers endothelial dysfunction and vascular remodeling processes. This pathophysiological condition leads to structural abnormalities at the macrovascular level (e.g., aorta) and functional and morphological changes at the microvascular level [15]. In particular, oxidative stress and the chronic release of inflammatory cytokines, especially TNF-α and IL-6, promote extensive endothelial dysfunction in axSpA patients [16,17]. In addition to systemic inflammatory activity, oxidative stress plays an important role in the development of microvascular changes in axSpA. Increased levels of reactive oxygen species (ROS) contribute to endothelial damage, disrupt vascular integrity, and impair capillary autoregulation. Chronic inflammation-induced oxidative stress is associated with capillary thinning, vascular remodeling and altered retinal perfusion. In addition, endothelial dysfunction, characterized by decreased nitric oxide bioavailability and persistent inflammatory stimulation, further exacerbates microvascular impairment by promoting vascular instability and impairing the distribution of oxygen. These mechanisms may underlie the observed reductions in SCP and DCP densities in axSpA patients, irrespective of treatment modality [11,16,17].

While anti-TNF agents are effective in reducing systemic inflammation, their ability to fully reverse endothelial damage caused by oxidative stress remains uncertain. Although TNF-α inhibition may attenuate inflammation-induced endothelial activation, pre-existing vascular remodeling may limit the extent of microvascular recovery. Our study showed a significant difference in choriocapillaris density between patients receiving anti-TNF therapy and those taking only NSAIDs. However, the lack of significant differences in SCP and DCP density suggests that the control of systemic inflammation alone may not be sufficient to prevent or reverse microvascular changes. Further research is needed to investigate whether interventions targeting oxidative stress could provide additional microvascular protection in axSpA [12,13].

One of the best-known pathologies at the macrovascular level is aortitis [18]. This disease can manifest as aneurysmal dilatation, fibrotic changes, and the thickening of vessel walls, particularly in the segments of the aortic root and ascending aorta. In contrast, microvascular changes often take a subclinical course and are difficult to detect at an early stage using conventional methods [14,19]. Particularly in the retina, which is a metabolically active tissue due to its high oxygen demand, these subclinical inflammatory processes can cause microvascular abnormalities that are detectable with OCT-A [5].

Microvascular involvement in AxSpA patients is not limited to the narrowing of the vascular lumen but also leads to disturbances such as reduced capillary perfusion, the loss of the homogeneity of blood flow, and an irregular oxygen distribution [20]. It has been reported that these changes can partially regress when inflammation is suppressed at a clinical or subclinical level [21]. Anti-TNF agents, which are commonly used in the treatment of axSpA, may also have beneficial effects at the microvascular level by reducing TNF-α mediated endothelial activation and inflammatory cell infiltration. While the efficacy of anti-TNF treatments on macrovascular complications (e.g., aortitis) has been more thoroughly investigated in the literature, few data are available on their effects at the microvascular level [22,23,24].

In this study, a statistically significant difference was found at the choriocapillaris level between patients receiving anti-TNF therapy and those taking only NSAIDs. This finding may suggest that the effect of anti-TNF agents may be limited to microvascular structures and that simply controlling systemic inflammation may not be sufficient to affect microvascular changes in the superficial and deep capillary plexus. The lack of a significant relationship between ASDAS-CRP scores, which assess disease activity, and retinal capillary density suggests that retinal microcirculatory disturbances in axSpA may be more related to cumulative effects as a function of disease duration. Accordingly, we found that there were differences at the microvascular level in axSpA patients compared to healthy patients, but there was no significant difference depending on the medication used.

Although recent research on OCT-A findings in rheumatic diseases is increasing, data are still limited. For example, Ayar et al. (2021) showed that the capillary plexus density (SCP and DCP) assessed with OCT-A was significantly lower in patients with rheumatoid arthritis compared to healthy controls. Furthermore, in a study investigating the relationship between disease activity and capillary density, a negative correlation was found between DAS28 scores and radial peripapillary capillary density [25]. Yener et al. (2022) reported that the density of deep DCP was significantly decreased in patients with primary Sjögren’s syndrome compared to a healthy control group. In our study, we similarly observed a decrease in DCP VD [26]. This may suggest that the inflammatory process observed in both diseases may be the cause. Similarly, Çalışkan et al. (2020) showed that the capillary plexus density (SCP and DCP) assessed by OCT-A was significantly decreased in axSpA patients compared to healthy controls. Furthermore, a negative correlation was found between disease duration and DCP density, but no significant relationship between disease activity (BASDAI) and capillary density was found [27]. In our study, we also observed a negative correlation between DCP VD and disease duration, and we also found no significant relationship between disease activity (ASDAS-CRP) and VD.

These results suggest that microvascular changes in AxSpA may be associated with the cumulative effects of inflammation and that retinal microvascular changes in rheumatic diseases can be detected at the subclinical level with OCT-A, which may serve as a tool to assess inflammatory processes.

## 5. Limitations

In this study, ASDAS was used to assess disease activity in axSpA. Although inflammation markers such as CRP and blood sedimentation, which are included in ASDAS, were present in the study, the effects of biological variations in inflammation and other possible factors were not investigated in detail. The relationships between disease duration and the OCT-A findings were evaluated, but a larger sample size is needed to improve the reliability of these results. In addition, among the patients taking medication, there were those receiving anti-TNF therapy and those taking only NSAIDs. This made it difficult to clearly distinguish the contribution of treatment effects to the OCT-A findings. Finally, this study was conducted in a limited patient population, and further studies with larger sample groups are needed to increase the generalizability of the results. This study has provided important insights as it is the first study to determine the differences between anti-TNF and NSAIDs by creating a subgroup in patients with axSpA and the impact of 5-year outcomes of anti-TNF treatment on microvascular perfusion.

## 6. Conclusions

In conclusion, we found that retinal and choroidal microvascular changes that may be caused by the inflammatory process induced by axSpA can be detected with OCT-A. We found that these findings increased with increasing disease duration and that the drug option used affected the choriocapillaris’s vasculature. The results of this study represent a novel application of OCT-A and demonstrate its potential in the context of axSpA. This imaging technique may facilitate the identification of different stages of retinal microvascular changes in axSpA and contribute to the development of new classification systems for the vascular involvement associated with the disease. In addition, vascular remodeling detected by OCT-A could serve as a marker for monitoring disease progression and evaluating the efficacy of systemic treatments, including NSAIDs and biologic therapies such as anti-TNF agents. Retinal vascular changes may also provide insight into systemic vascular conditions, supporting the development of innovative models to assess disease progression or systemic complications. Finally, investigating the relationship between changes in retinal microcirculation and the extent of joint or spinal damage in axSpA could offer valuable perspectives.

## Figures and Tables

**Table 1:** Demographic and ocular parameters of the subjects. TNF: Tumor necrosis factor, NSAID: Non-steroidal anti-inflammatory drugs, ASDAS-CRP: Ankylosing Spondylitis Disease Activity Score—C-Reactive Protein, IOP: Intraocular pressure, SE: Spherical equivalent, D: Diopters, VA: Visual acuity, logMAR: Logarithm of the minimum angle of resolution, HLA: Human leukocyte antigen. Values are written as mean value ± standard deviation and (minimum–maximum) value in the bottom row. Statistically significant values are indicated in bold.

	Anti TNF Group (*n*:26)	NSAID Group (*n*:22)	Control (*n*:33)	*p* Value
Age (years)	46.77 ± 9 (32–70)	42 ± 12 (20–62)	44.3 ± 13 (28–73)	0.397 *
IOP (mmHG)	14.36 ± 5.1 (8–22)	16.1 ± 3.6 (10–23)	15.2 ± 4 (8–22)	0.161 **
SE (D)	−0.33 ± 1.4	−0.92 ± 0.76	−1 ± 1.4	0.469 **
Axial Length (mm)	23.35 ± 0.76 (21.96–24.79)	23.36 ± 0.16 (21.82–26.46)	23.38 ± 0.72 (21.86–25)	0.087 **
HLA B27 (+)	17	9	-	0.016 ***
Radiography (+)	24	16	-	0.010 ***
Duration of Drug Usage (Years)	13 ± 8.8 (5–45)	9.27 ± 6.9 (5–33)	-	0.71 **
Duration of Disease (Years)	17.9 ± 7 (7–47)	9 ± 7.5 (5–35)		<0.001 *

* ANOVA. ** Kruskal–Wallis H test. *** Pearson’s chi-square test.

**Table 2:** Comparison of the retina and RNFL thicknesses between groups. TNF: Tumor necrosis factor, NSAID: Non-steroidal anti-inflammatory drugs, RNFL: retinal nerve fiber layer, Cent: central, Temp: temporal, Sup: superior, Nas: nasal, Inf: inferior, µm: micron. Values are written as mean value ± standard deviation and (minimum–maximum) value in the bottom row. Statistically significant values are indicated in bold.

	Anti TNF Group	NSAID Group	Control	*p* Value
Retina Cent (µm)	252 ± 23	244 ± 22	246 ± 39	0.044 *
Temp (µm)	258 ± 27	248 ± 24	246 ± 39	0.747 *
Sup (µm)	299 ± 17	296 ± 20	297 ± 29	0.521 *
Nas (µm)	308 ± 24	313 ± 17	309 ± 25	0.066 *
Inf (µm)	314 ± 19	314 ± 14	302 ± 31	0.858 *
Mean (µm)	304 ± 22	306 ± 21	303 ± 28	0.533 *
RNFL Cent (µm)	10 ± 6	11 ± 5	9 ± 8	0.068 *
Temp (µm)	23 ± 5	27 ± 12	26 ± 15	0.793 *
Sup (µm)	29 ± 5	33 ± 8	31 ± 8	0.192 *
Nas (µm)	27 ± 6	30 ± 9	25 ± 7	0.024 *
Inf (µm)	27 ± 7	33 ± 13	29 ± 10	0.023 *
Mean (µm)	23 ± 3	26 ± 6	24 ± 7	0.004 *

* Kruskal–Wallis H test.

**Table 3:** Comparison of the choroid and GCL thicknesses between groups. TNF: Tumor necrosis factor, NSAID: Non-steroidal anti-inflammatory drugs, GCL: ganglion cell layer, Cent: central, Temp: temporal, Sup: superior, Nas: nasal, Inf: inferior, µm: micron. Values are written as mean value ± standard deviation and (minimum–maximum) value in the bottom row.

	Anti TNF Group	NSAID Group	Control	*p* Value
Choroid Cent (µm)	263 ± 72	307 ± 50	278 ± 79	0.374 *
Temp (µm)	255 ± 69	298 ± 78	271 ± 76	0.472 *
Sup (µm)	287 ± 74	318 ± 55	271 ± 85	0.214 *
Nas (µm)	267 ± 70	286 ± 51	265 ± 84	0.755 *
Inf (µm)	257 ± 76	306 ± 46	272 ± 85	0.331 *
Mean (µm)	266 ± 58	303 ± 52	271 ± 78	0.406 *
GCL Cent (µm)	23 ± 3	26 ± 6	24 ± 7	0.004 *
Temp (µm)	56 ± 13	52 ± 13	48 ± 13	0.005 *
Sup (µm)	84 ± 8	81 ± 11	84 ± 16	0.006 *
Nas (µm)	88 ± 11	89 ± 8	89 ± 14	0.485 *
Inf (µm)	90 ± 10	90 ± 7	85 ± 15	0.371 *
Mean (µm)	87 ± 11	84 ± 12	88 ± 16	0.002 *

* Kruskal–Wallis H test.

**Table 4:** Comparison of the FAZ area, superficial, deep capillary plexus, and choriocapillaris vascular density between the groups. TNF: Tumor necrosis factor, NSAID: Non-steroidal anti-inflammatory drugs, Cent: central, Temp: temporal, Sup: superior, Nas: nasal, Inf: inferior, SCP: superficial capillary plexus, VD: vessel density, DCP: deep capillary plexus, CC: choriocapillaris. Values are written as mean value ± standard deviation and (minimum–maximum) value in the bottom row. Statistically significant values are indicated in bold.

	Anti TNF Group (1)	NSAID Group (2)	Control (3)	*p* Value (1–2–3)	*p* Value (1–2)	*p* Value (1–3)	*p* Value (2–3)
FAZ (mm^2^)	339 ± 80	341 ± 144	295 ± 69	0.015 *	0.542 ***	0.002 ***	0.111 ***
SCP VD—foveal central (%)	18 ± 3	21 ± 8	21 ± 5	0.033 *	0.124 ***	0.007 ***	0.664 ***
temp (%)	47 ± 3	46 ± 2	46 ± 3	0.107 *	-	-	-
sup (%)	49 ± 3	48 ± 3	45 ± 4	<0.001 **	0.253 ‡	**<0.001 ‡**	**<0.001 ‡**
nas (%)	46 ± 4	46 ± 3	45 ± 4	0.065 *	-	-	-
inf (%)	46 ± 4	46 ± 4	43 ± 5	<0.001 *	0.657 ***	<0.001 ***	<0.001 ***
mean (%)	41 ± 2	41 ± 2	40 ± 2	<0.001 *	0.385 ***	0.001 ***	<0.001 ***
DCP VD—foveal central (%)	17 ± 4	18 ± 9	23 ± 8	<0.001 *	0.691 ***	<0.001 ***	<0.001 ***
temp (%)	47 ± 3	47 ± 3	48 ± 3	<0.001 *	0.605 ***	0.268 ***	0.156 ***
sup (%)	52 ± 4	51 ± 3	49 ± 4	<0.001 *	0.608 ***	<0.001 ***	<0.001 ***
nas (%)	49 ± 3	49 ± 3	48 ± 5	0.082 *	-	-	-
inf (%)	50 ± 4	50 ± 4	45 ± 4	<0.001 *	0.734 ***	<0.001 ***	<0.001 ***
mean (%)	43 ± 2	43 ± 2	42 ± 2	0.465 *	-	-	-
CC VD—foveal central (%)	51 ± 5	53 ± 3	49 ± 4	<0.001 **	0.071 ‡	0.057 ‡	**<0.001 ‡**
temp (%)	54 ± 2	54 ± 2	53 ± 3	0.148 *	-	-	-
sup (%)	53 ± 3	53 ± 2	50 ± 5	<0.001 *	0.135 ***	0.001 ***	<0.001 ***
nas (%)	54 ± 2	54 ± 2	53 ± 3	0.046 *	0.567 ***	0.063 ***	0.025 ***
inf (%)	53 ± 3	54 ± 4	49 ± 4	<0.001 *	0.221 ***	<0.001 ***	<0.001 ***
mean (%)	53.2 ± 2.4	53.9 ± 1.9	51 ± 3	<0.001 *	0.038 ***	<0.001 ***	<0.001 ***

* Kruskal–Wallis H test. ** ANOVA. *** Mann–Whitney U test. ‡ Tukey’s HSD test.

## Data Availability

The raw data supporting the conclusions of this article will be made available by the authors on request.
